# Understanding Physical Resilience in Aging: Insights from Intrinsic Capacity Domains

**DOI:** 10.1093/geroni/igaf122.2480

**Published:** 2025-12-31

**Authors:** Hyeon Jung Heselton, Julie Blaskewicz Boron

**Affiliations:** University of Nebraska Lincoln, Lincolne, Nebraska, United States; University of Nebraska Omaha, Omaha, Nebraska, United States

## Abstract

Maintaining a high quality of life is a central goal for aging adults, as it directly influences their well-being, independence, and overall health in later life. Older adults experience reduced physical resilience (PR), affecting their ability to recover from physical stressors. The World Health Organization’s concept of intrinsic capacity (IC) captures functional potential and resilience across multiple domains. This study explored physical resilience (PR; loss of balance) through three physical IC domains: vitality (handgrip strength), sensory (hearing and vision), and locomotion (walking speed). It also investigated whether age moderated the IC and PR relationship. Forty-six healthy middle-aged (n = 25; 52% female; 55.8±7.02yrs) and older (n = 21; 47.6% female;70.14±3.55yrs) adults completed five standing trials on a force platform with perturbations to measure PR. Results indicated that higher handgrip strength and faster walking speed were significantly associated with lower loss of balance (F(1,44)=6.03, p = 0.018, R²=0.12; F(1,44)=4.88, p = 0.032, R²=0.10, respectively). Hearing and vision did not predict PR. Age did not significantly influence the IC and PR relationship. Results revealed consistent results from previous research that handgrip strength and walking speed are strong predictors of PR, whereas sensory IC may be more associated with compensatory mechanisms such as proprioception and vestibular function in maintaining balance. It is well-known that biological changes with age affect physical functions, but a more comprehensive understanding of IC, along with other factors (physical activity, diet, sleep) and its interaction with aging, is essential for better insight into functional resilience in later life.

